# Foreign Correspondence

**Published:** 1839-11-02

**Authors:** 


### FOREIGN CORRESPONDENCE.

From a letter of a physician who formerly held a distinguished appointment in the Texan army, to a medical friend in this city, we learn the following interesting particulars respecting the yellow fever of Galveston. It would seem that this disease has included a very large number of places in the circle of its ravages; in some it has presented signs of great malignancy, while in others, as at Galveston, it was extremely limited. An accurate account of the progress of the fever along the different parts of the coast, would be one of the most interesting medical documents for the study of epidemics:

"About the 1st of October the yellow fever made its appearance on the Strand—the street bordering the harbour—terminating many cases with unequivocal black vomit. The disease still prevails with a good deal of severity, though somewhat abated—from three to six dying daily. When the epidemic was at its height, the thermometer ranged at mid-day from 84° to 88°. I will not trouble you with minute details at this time, especially as I am preparing an account of the disease. I have made four post-mortem examinations; the details of these will be given at length.

"In all the examinations we have found the liver fawn-coloured, externally and internally,—of its ordinary firmness of structure,—no bile in its parenchyma, and but a small quantity of *very dark* and *viscid* bile in the gall-bladder. But the stomach manifested unequivocal evidences of the most violently diseased condition. It, in all the cases, contained black vomit, as well in one who had not vomited it previously to death, as in the others who had. The flocculi, which swim in the black, turbid liquor, were found adherent to the mucous coat. This was *awfully injected* in the four cases about the cardiac orifice; in all its remaining portions greatly thickened. The duodenum, juncture of ileum and cæcum, and other portions of intestines, manifested evidences of considerable disease, and contained no bile nor 'bilious matters.' But the stomach exhibited the 'head and front of the offending,'—soon after death all turned yellow. No case has occurred off the Strand, i. e. the infected district."

